# Effectiveness of psychological interventions for reducing depressive symptoms among women experiencing perinatal depression: a systematic review

**DOI:** 10.1016/j.xagr.2026.100654

**Published:** 2026-06-01

**Authors:** Abdissa Boka, Debela Gela, Jembere Tesfaye

**Affiliations:** 1 Department of Emergency (Boka), Critical and Chronic care Nursing, School of Nursing and Midwifery, College of health Sciences, Addis Ababa University, Addis Ababa, Ethiopia; 2 Department of Medical Surgical Nursing (Gela), School of Nursing and Midwifery, College of Health Sciences, Addis Ababa University, Addis Ababa, Ethiopia; 3 Department of Midwifery (Tesafye), School of Nursing and Midwifery, College of Health Sciences, Addis Ababa University, Addis Ababa, Ethiopia

**Keywords:** cognitive behavioral therapy, counseling, interpersonal therapy, low and middle income countries, psychological interventions, psychotherapy, perinatal depression

## Abstract

**Background:**

Psychological interventions are well established as effective treatments for perinatal depression, with strong evidence supported by randomized controlled trials carried out in diverse cultural contexts and in settings with limited resources. Approaches such as cognitive behavioral therapy, problem-solving therapy, interpersonal therapy, and community based models that rely on task-sharing have repeatedly shown meaningful reductions in depressive symptoms and notable improvements in maternal functioning and overall well-being. These interventions are especially important in low- and middle- income countries (LMICs) where access to specialist mental health professionals is seriously limited. Evidence demonstrating that appropriately trained non-specialist including community health workers (CHW), Peers, and lay counselors can successfully deliver this intervention within primary health care and community platforms highlighting their practicality and effectiveness. Consequently, task-sharing strategies are therefore considered a promising and sustainable approach for reducing the treatment gap for perinatal depression in resource-constrained contexts.

**Objective:**

This systematic review aimed to evaluate the effectiveness of psychological interventions in reducing depressive symptoms among women experiencing perinatal depression in low and middle income countries.

**Study Design:**

Studies were eligible if they evaluated task-shared or lay-delivered psychological interventions for perinatal depression among pregnant or postpartum women aged 18 years and older in LMICs. Only randomized controlled trials or cluster randomized controlled trials were included. Participants were required to have screened positive for depressive symptoms or to have been diagnosed with major depressive disorder using validated screening or diagnostic instruments. Studies that solely addressed severe mental illness necessitating inpatient psychiatric treatment lacked a comparison group or did not report outcomes related to depression were excluded.

**Methods and information sources:**

Major electronic databases commonly utilized in health research were thoroughly and systematically searched, with the most recent search conducted in Month/Year. Only studies published in English were considered. The study selection was carried out in staged manner according to predetermined eligibility criteria, with independent review of titles, abstracts, and full-text articles. The methodological quality and risk of bias of included studies were evaluated using standardized appraisal tools and relevant data were collected using a structured and piloted data extraction form. Due to substantial heterogeneity in intervention content, delivery modes, settings, and outcome measures, a narrative synthesis was undertaken rather than a meta-analysis. The level of confidence in the evidence for the main outcomes was evaluated, and no substantial deviations from the predetermined methodological plan were observed.

**Results:**

Eleven studies met the inclusion criteria, comprising ten randomized controlled trials and one formative mixed-methods study, with a total sample of **4809** perinatal women from Pakistan, South Africa, and Tanzania. Interventions primarily involved task-shared psychological therapies delivered by peers, community health workers, or lay counsellors, often based on cognitive behavioral or problem-solving principles. Study quality was mixed, with generally low risk of bias in well-conducted trials but important limitations related to heterogeneity of outcome measures, limited reporting on cost-effectiveness, and incomplete effectiveness data in formative and protocol-based studies. Technology-assisted, peer-delivered interventions demonstrated similar or greater reductions in depressive symptoms and higher remission rates compared with standard care. Conversely, certain task-shared interventions did not demonstrate a statistically meaningful advantage when compared with enhanced usual care. Overall, the certainty of evidence ranged from low to moderate across outcomes.

**Conclusion:**

Task-shared psychological interventions for perinatal depression appear feasible and acceptable in low-resource settings. From the review findings the availability of technology assisted interventions that delivered by peers can achieve similar clinical outcomes that comparable to interventions delivered by mental health professionals. Nevertheless, both effectiveness and cost-efficiency differ depending on context, level and intervention intensity, as well as the capacity of health system in which they are implemented. Additional rigorously designed studies, incorporating standardized outcome measures, extended follow-up durations, and comprehensive economic evaluations are needed to reinforce the existing evidence and to inform policy decisions and large-scale implementation strategies.

**Systematic review registration:**

PROSPERO, CRD420261285318.

**AJOG MFM at a Glance**

**Why was this study conducted?**

To evaluate the effectiveness of task-shared psychological interventions for perinatal depression in low- and middle-income countries (LMICs), where specialist mental health services are limited. To synthesize updated evidence on community- and digitally delivered therapies integrated into routine maternal care.

**Key findings?**

Across 11 randomized and cluster randomized trials (n = 4809), most task-shared Cognitive Behavioral Therapy, Interpersonal psychotherapy, and Problem Solving Therapy based interventions significantly reduced depressive symptoms. Digitally supported, peer-delivered adaptations of the Thinking Healthy Program achieved remission rates comparable or superior to standard health worker delivery.

**What does this add to what is known?**

Provides updated synthesis demonstrating that digitally supported, peer-delivered psychological interventions are feasible, safe, and scalable in LMICs. Highlights contextual variability in effectiveness and cost-effectiveness, underscoring the need for standardized outcomes and long-term evaluation.

## Introduction

Perinatal depression, which refers to depressive episodes occurring during pregnancy or within the first year following delivery, constitutes a major public health concern worldwide with substantial effects on women, infants, families, and healthcare systems. Perinatal depression is one the leading mental health problems that affects women of childbearing age with global estimate of the 10% to 20% prevalence during this period. The rate of perinatal depression is notably greater in low- and middle-income countries (LMICs), reflecting significant inequalities in both the burden of mental illness and the availability of mental health services.^1^, ^2^, ^3^, ^4^ If this condition is not addressed, it can lead to serious adverse outcomes for mothers, such as reduced ability to carry out daily functioning, decreased quality of life, and higher likelihood of persistent or recurrent depression. Additionally, it can have adverse effects on infants; including preterm birth, low birth weight, disrupted mother–infant bonding, and long-term cognitive, emotional, and behavioral challenges.^5^, ^6^, ^7^, ^8^

While antidepressants medications are effective for depressive disorders, their use during perinatal period is often limited by safety concerns related to pregnancy, lactation, women preferences, social stigma, and inadequate availability of specialist mental health services predominantly in resource-limited settings.^9^^,^^10^ Consequently, non-pharmacological approaches are commonly endorsed as a primary treatment option for women suffering from mild to moderate perinatal depression and as adjunctive therapies for more severe cases.^11^^,^^12^ These interventions are particularly relevant in health system where access to pharmacotherapy is unavailable, unacceptable, or not fully embedded within standard maternal services.

As different evidences from various research demonstrates the psychological interventions effectiveness for perinatal depression, encompassing approaches such as cognitive behavioral therapy (CBT), interpersonal therapy (IPT), problem-solving therapy (PST), behavioral activation, mindfulness-based techniques, and other related psychosocial interventions.^13^, ^14^, ^15^ The findings from systematic reviews and meta-analyses indicate that these interventions are effective in markedly lowering the severity of depressive symptom and enhancing maternal psychosocial wellbeing during pregnancy as well as in the postpartum period.^16^^,^^17^ The effectiveness of Psychological interventions has been demonstrated through multiple modes of delivery, such as one to one or individual and group-based approaches, in-person and digital platforms, as well as hybrid approaches that combines different methods of care.^18^, ^19^, ^20^

As the evidence of the latest studies indicates, there is growing interest in task-sharing and community-based psychological interventions provided by non-specialist personnel, including nurses, midwives, community health workers, peers, and trained lay counsellors. Such strategies are especially crucial in LMICs, where limited availability of mental health specialists remains a significant obstacle to accessing care.^21^^,^^22^ Landmark trials have shown that brief, well-structured psychological interventions embedded whining routine maternal and primary healthcare services can produces clinically meaningful improvements in perinatal depressive symptoms.^23^, ^24^, ^25^ These results underscore the practicability, scalability, and potential economic efficiency of task-shared psychological interventions integrated into existing healthcare systems.

Notwithstanding the expanding body of evidence, considerable uncertainty persists. The literature on psychological interventions for perinatal depression is marked by substantial heterogeneity, encompassing wide variation in intervention components, intensity, timing of delivery (antenatal versus postnatal), provider type, settings, outcome measures, and overall methodological quality.^26^^,^^27^ In addition, a recent study has introduced culturally tailored interventions, trauma-informed models, and digital or technology-assisted delivery platforms, further complicating the evidence landscape.^28^, ^29^, ^30^, ^31^ Collectively, these developments highlight the necessity for an updated and comprehensive synthesis to determine which psychological interventions are effective, for whom, and under what contextual conditions.

Although several previous systematic reviews have examined psychological interventions for perinatal depression; however, many focused on specific intervention types, specialist-delivered models, or high-income country settings. Others predate the rapid expansion of task-sharing approaches and digitally supported interventions in LMICs. A preliminary search of PROSPERO, PubMed, Scopus, Web of Science, the Cochrane Database of Systematic Reviews, and JBI Evidence Synthesis identified no current or ongoing systematic reviews that comprehensively address the effectiveness of psychological interventions for perinatal depression in low and middle income countries. Accordingly, this systematic review evaluates the effectiveness of psychological interventions to reduce symptoms of perinatal depression at healthcare and community settings, with particular emphasis on task-sharing approaches. These interventions seek to alleviate depressive symptoms by enhancing coping capacity, addressing maladaptive thoughts and behaviors, promoting involvement in meaningful activities and enhancing interpersonal relationships and social support system.

## Objective

The objective of this systematic review is to evaluate the effectiveness of psychological interventions within the primary healthcare settings for the treatment of perinatal depression throughout the perinatal period, compared with usual care or enhanced usual care. The primary outcome of interest is the reduction in depressive symptom severity while secondary outcomes encompass improvement in anxiety, functional status, quality of life, social support, maternal well-being, and infant-related outcomes.

*Research Question:* What is the effectiveness of psychological interventions to reduce symptoms of perinatal depression among pregnant and postnatal women of low and middle income countries?

## Methods

### Eligibility criteria and intervention description

The eligibility of Studies was predefined based on the population, intervention, comparator and outcomes and study design (PICOs) framework. The population of interest comprised women aged 18 years or older in LMICs during perinatal period who met diagnostic or screening criteria for perinatal depression including major depressive episode identified by using validated instruments (Structural Clinical Interview Diagnostic statistical manual (SCID), Edinburgh Postnatal Depression Scale (EPDS), Patient health Questionnaire (PHQ-9), Hamilton Depression Rating Scale (HDRS-17). As well as trials enrolling specific sub-populations, such as women living with HIV were eligible provided depressive symptoms were explicit inclusion criteria. However, studies focuses on women with severe mental illness requiring inpatient psychiatric care were excluded.

Eligible interventions included structured psychological or psychosocial approaches aimed at reducing depressive symptoms among women experiencing perinatal depression. The review primarily focused on interventions delivered through task-sharing (task-shifting) models, in which evidence-based psychological therapies are provided by non-specialist providers such as community health workers, lay counsellors, peers with lived experience, nurses, or midwives—within primary healthcare or community settings. These providers typically receive standardized training in specific therapeutic approaches and ongoing supervision to ensure fidelity, quality, and consistency of intervention delivery. This model is particularly relevant in low- and middle-income countries (LMICs), where the availability of specialist mental health professionals is limited, and aims to improve access, affordability, and cultural relevance of mental health care.

The interventions included in this review were grounded in established therapeutic frameworks, most commonly Cognitive Behavioral Therapy (CBT) and Problem-Solving Therapy (PST). CBT-based interventions focus on identifying and modifying maladaptive thoughts and behaviors, promoting behavioral activation, and strengthening coping mechanisms, while PST emphasizes structured identification of life stressors and development of practical problem-solving skills. In addition, several interventions incorporated technology-assisted delivery models, such as mobile applications or digital platforms, to support non-specialist providers, enhance adherence to therapeutic protocols, and improve scalability of the interventions.

To be eligible for inclusion, studies were required to report at least one depression-related outcome, including remission from major depressive disorder, treatment response, or change in depressive symptom severity measured using validated instruments. Secondary outcomes such as anxiety, functional status, quality of life, social support, cost-effectiveness, and infant-related outcomes were extracted where available but were not mandatory for study inclusion.

Comparator conditions varied across studies and were categorized as routine or standard care, usual care (UC), and enhanced usual care (EUC). Routine or standard care generally refers to basic maternal health services, including antenatal and postnatal care, without structured mental health screening or psychological support. Usual care involves the standard clinical pathway, where individuals identified with depression may be referred for further care but typically receives no structured follow-up or dedicated psychological intervention. Enhanced usual care represents a more comprehensive comparator, often including additional elements such as training of healthcare providers on mental health screening results, provision of informational resources, and basic psychosocial support (eg, WHO mhGAP-based care), while remaining less intensive than the structured psychological interventions evaluated.

Eligible study designs included randomized controlled trials and cluster randomized controlled trials conducted in low- and middle-income countries, reflecting the review’s focus on health systems with limited specialist mental health resources. Formative, qualitative, or mixed-methods studies without comparative effectiveness data were excluded from the quantitative synthesis but were considered descriptively where they informed intervention development or implementation. Studies were required to include a minimum follow-up period extending into the postnatal phase, typically up to 3 months postpartum or longer. With respect to report characteristics, peer-reviewed journal articles published in English and published since January 2005 to December 2025 were eligible. Conference abstracts, unpublished manuscripts, and grey literature were excluded as they were not retrieved through the search strategy. For the narrative synthesis, interventions were categorized by their delivery model (eg, peer-delivered, technology-assisted, or health worker-led). These were compared against specific control conditions, which were further sub-grouped into “usual care” (routine maternal services) and “enhanced usual care” (routine services plus additional screening or referral resources and outcome domain (depression remission, symptom reduction, and functional outcomes), consistent with the review objectives. These classifications facilitated systematic comparison of intervention effectiveness across populations and delivery models. Limitation to randomized study designs and inclusion of postnatal outcome measures were applied to maintain methodological rigor and ensure clinical relevance.

### Information sources

A comprehensive literature search was undertaken across multiple electronic databases, including PubMed/MEDLINE, Cochrane Library, Research4Life, and Google Scholar. Searches were finalized in January 2026 encompassing studies published in database inception up to that date. In PubMed the MEDLINE interface was utilized a combination of MeSH terms and free-key text words, whereas the Cochrane Library and Research4Life were searched using title, abstract, and keyword fields. Google Scholar was used to identify additional relevant and grey literature. The search was restricted to articles published in English. In addition, reference lists of all included studies and relevant systematic reviews on perinatal depression and psychological interventions were manually screened to identify further eligible studies. Forward and backward citation searching was undertaken using Google Scholar to capture any additional relevant publications. No direct contact was made with study authors, organizations, or intervention developers, and no journal hand searching or conference proceedings were consulted.

### Search strategy

A PICO-style conceptual framework search strategy was developed by combining terms related to perinatal women, psychological interventions, and depression outcomes, with an additional filter for low- and middle-income countries. Searches were conducted using both controlled vocabulary MeSH terms and free-text keywords were used for search by combining with Boolean operators. In PubMed/MEDLINE, searches were run line by line using MeSH terms and title/abstract fields, while simplified keyword sequences were applied in the Cochrane Library, Research4Life, and Google Scholar. Published MeSH terms and commonly used keywords from prior systematic reviews in perinatal mental health informed the strategy, with minor adaptations to suit each database interface. No text-mining, natural language processing, automatic search translation tools, formal validation sets, or peer-review processes were employed.

### Selection process

All records identified were imported into a reference manager EndNote X9 and duplicates removed. Two reviewers independently screened titles/abstracts and then full text against the inclusion criteria, with disagreement resolved by consensus. Study investigators were not contacted to obtain or clarify additional information, and no translations were required as only English-language publications were eligible.

### Data items

The extracted data predefined for outcome domains including depressive symptom severity or remission, anxiety symptoms, functional disability, quality of life, social support, and selected maternal and infant health outcomes where reported. Outcomes were measured at baseline and short-term follow-up (around 3 months postpartum), prioritizing validated instruments, with reductions in depressive symptoms or remission considered the primary outcomes. Alongside outcomes, data on study, participant, and intervention characteristics, follow-up, sample size and attrition were extracted using standardized form. Missing or unclear information was recorded as not reported, with no assumptions made.

### Risk of bias and effect

Risk of bias was assessed using JBI critical appraisal tools with two reviewers conducting independent evaluation and resolving any disagreements by the third reviewer without modifying existing tools or contacting study authors.

### Synthesis methods

Studies were considered eligible for synthesis based on similarity in population, intervention type, comparator, and outcome measures, in accordance with the review’s predefined inclusion criteria. As a result of substantial clinical and methodological heterogeneity, a narrative synthesis approach was used. The data completeness and consistency were checked and results were summarized in tables presenting key study characteristics, interventions, outcomes, and main findings. Statistical synthesis and meta-analysis were not performed, nor were data transformation or sensitivity analyses undertaken. Instead, heterogeneity was explored narratively through comparisons of studies by intervention type, setting, delivery agent, and lengths of follow-up.

### Reporting bias assessment

Reporting bias was not formally assessed using statistical or graphical methods because the review not includes meta-analysis as well as the review considered a few studies. In this regard, Risk of bias due to missing the result was assessed narratively and two reviewers independently compared reported outcomes with study protocols and methods, where available, to check completeness of outcome reporting, resolving disagreements by consensus. No formal tools, automation, or contact with study authors were used.

### Certainty assessment

Using the JBI critical appraisal and certainty assessment approach, the methodological quality, appropriate study design, clearly defined outcomes, and adequate statistical analyses of the individual studies was assessed with the overall Grading of Recommendations Assessment, Development and Evaluation (GRADE) of the primary outcomes was judged to be moderate. Nevertheless, the certainty was downgraded due to risk of bias in blinding and allocation concealment in several studies, and moderate heterogeneity across study results. Overall, the evidence suggests that the observed effects are likely to be close to the true effect, but further well-conducted studies may have an important impact on confidence in the estimate.

## Results

### Study selection process

The study selection process was documented using a PRISMA flow diagram. After duplicates were removed, records were screened based on title and abstract, and the full texts of potentially eligible studies were evaluated against predefined inclusion criteria (such as inappropriate study design, population, intervention, or outcomes). Screening was performed manually by reviewers without automation, and no ongoing studies or updates of previous reviews were identified. From total search results, eleven studies met the inclusion criteria with description of ten completed randomized controlled trials and one formative mixed-methods intervention development study. The review focused on low- and middle-income countries, including studies from Pakistan = 3, Iran = 2, South Africa = 2, Tanzania = 1, Kenya = 1 Jordan = 1 and Ethiopia = 1 with sample sizes ranging from 24 to 1154 participants with total sample size of 4809, all of which assessed task-shared psychological interventions for perinatal depression delivered by lay peers, community health workers, or primary health care workers, with some incorporating digital technology support.

Report the number of papers identified by the search strategy and the number of papers included and excluded at each stage of the study selection process. Refer to the figure containing the PRISMA flow chart (Figure).

**Figure:**
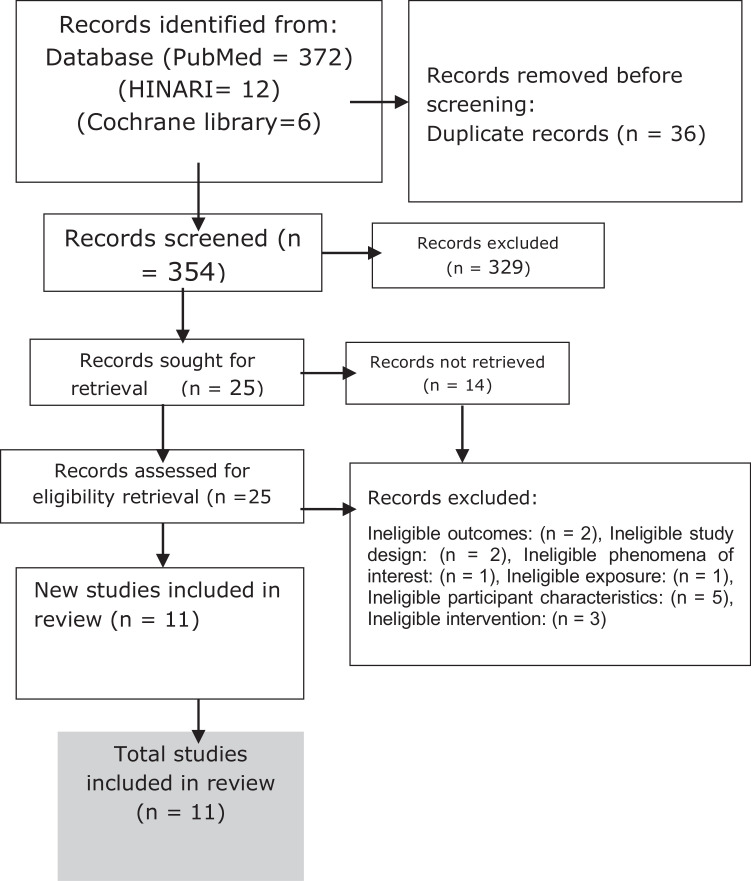
PRISMA 2020 flow diagram illustrating the study identification, screening, and selection process.

Where applicable, provide the citation details of all studies included after full-text assessment in the appendix, along with the key reasons for their inclusion. Similarly, where methodological quality assessment was conducted, include an appendix presenting the studies that met the quality criteria and the justification for their inclusion.

### Characteristics of psychological interventions in selected studies

Across studies, interventions were predominantly based on cognitive behavioral therapy (CBT) and problem-solving therapy (PST) principles with components such as psycho-education, behavioral activation, problem-solving, cognitive reframing, relaxation, and social support. Interventions were delivered individually, in groups, or in clinics across three to fourteen or more than it sessions during pregnancy and postpartum, using task-sharing via lay or community health workers, sometimes supported by digital technology for fidelity and scalability (Table 1). The excluded studies after full-text review were documented with reasons, including inappropriate design, populations not meeting perinatal depression criteria, non-psychological interventions, missing outcomes, or insufficient data with protocol without effectiveness of results.^3^ One mixed methods study which was included because it focuses on intervention development with evaluating clinical effectiveness (Table 1).

**Table 1:** Summary of characteristics of included studies.

Study	Country	Design	Sample size	Intervention provider	Comparator
Jannati et al.^32^	Iran	RCT	75	Self-guided mobile app (CBT-based)	Usual care
Abujilban et al.^33^	Jordan	RCT	100	Mental health therapist (telephone IPT)	Routine antenatal care
Yator et al.^34^	Kenya	Pilot RCT	24	Community Health Workers (group IPT)	Waitlist control
Rahman et al.^35^	Pakistan	Cluster RCT	903	Lady Health Workers	Enhanced routine care
Maselko et al.^36^	Pakistan	Cluster RCT	1154	Community Health Workers	Enhanced usual care
Rahman et al.^37^	Pakistan	Cluster RCT (non-inferiority)	980	Peers + digital app	LHW-delivered THP
Lund et al.^38^	South Africa	Cluster RCT	384	Community Health Workers	Enhanced usual care
Smith Fawzi et al.^39^	Tanzania	Cluster RCT	742	Lay community-based health workers (group-based)	Improved standard care (mhGAP)
Boisits et al.^40^	South Africa	Mixed-methods formative study	37	Community Health Workers	Not applicable
Rafat et al.^41^	Iran	RCT	108	Digital / app-based intervention	Routine prenatal care
Holmes et al.^42^	Ethiopia	Cluster RCT	302	Health Extension Workers (group CBT)	Standard postpartum care

### Intervention components and delivery of individuals’ studies

Overall, intervention characteristics and outcomes for both intervention and comparison groups were documented by using summary statistics extracted directly from the published articles (Table 2).

**Table 2:** Intervention components and delivery of individual studies.

Study	Core components	Sessions	Delivery mode	Setting
Jannati et al.^32^	CBT, behavioral activation, cognitive restructuring, problem-solving	8	Individual (mobile app)	Home
Abujilban et al.^33^	Interpersonal Psychotherapy (IPT)	7	Individual (telephone)	Home
Yator et al.^34^	Group IPT, emotional regulation, social support	8	Group	Clinic
Rahman et al.^35^	CBT, behavioral activation, problem-solving, psycho-education	Antenatal + postnatal	Individual	Home
Maselko et al.^36^	Enhanced CBT (THP Plus), behavioral activation	Longitudinal (pregnancy–36 mo)	Individual	Home
Rahman et al.^37^	CBT, behavioral activation, problem-solving (digital-assisted THP)	8	Individual	Home
Lund et al.^38^	PST, CBT, psycho-education, relaxation	6	Individual	Clinic/Home
Smith Fawzi et al.^39^	PST + CBT	Up to 14	Group	Clinic
Rafat et al.^41^	Health promotion education, behavioral change (app-based)	Not clearly stated	Individual (digital)	Home
Holmes et al.^42^	Group CBT, behavioral activation	4	Group	Health center

### Effects on perinatal depression outcomes: remission and symptom reduction

Evidence from the included trials demonstrated that the relative effectiveness of psychological interventions was significantly influenced by the type of comparator used.

Interventions Compared to Routine Usual Care: The most pronounced improvements in depressive symptoms and remission were observed in studies where interventions were compared against routine prenatal or postnatal care. For example, studies conducted in Iran and Jordan utilizing digital CBT-based applications and telephone-delivered interpersonal psychotherapy (IPT) reported significant reductions in depressive symptoms (*P*<.001) compared with standard clinical care. Similarly, in Ethiopia, group-based CBT integrated into routine health center services resulted in significant improvements in both depression and anxiety scores compared with standard postpartum care. These findings indicate that task-shared psychological interventions provide a clear clinical advantage in settings where structured mental health services are not part of routine care.

Interventions Compared to Enhanced Usual Care: In contrast, trials that utilized enhanced usual care (EUC) as a comparator where control groups received additional support such as screening feedback or basic psychosocial resources showed more variable or non-significant effects. For instance, a task-sharing counseling intervention in South Africa did not demonstrate a statistically significant difference in primary depression outcomes compared with EUC. Although small improvements in self-reported symptoms were observed, these did not reach clinically meaningful thresholds based on clinician-rated measures. These findings suggest that when usual care is strengthened with even modest mental health components, the relative effect size of structured psychological interventions may be reduced.

Technology-Assisted and Peer-Delivered Models: Notably, technology-assisted and peer-delivered interventions demonstrated high effectiveness. A large cluster randomized non-inferiority trial conducted in Pakistan showed that a digitally supported peer-delivered Thinking Healthy Program achieved a remission rate of 92.3% compared to 83.5% in the standard health worker–delivered group. The study applied appropriate non-inferiority methodology, including a pre-specified non-inferiority margin and one-sided confidence intervals corresponding to a stringent alpha level (*P*<.025), supporting the robustness of these findings. While these models show strong potential for scalability and high remission rates, their sustained long-term impact compared with standard interventions remains an area for further investigation (Table 3).

**Table 3:** Effects on depression outcomes.

Study	Primary outcome	Effect
Jannati et al.^32^	EPDS score	Significant reduction in depressive symptoms in intervention group (*P*<.001)
Abujilban et al.^33^	EPDS score	Significant improvement in antenatal depression compared with routine care (*P*<.001)
Yator et al.^34^	EPDS score	Significant reduction in depression scores; intervention feasible and acceptable
Rahman et al.^35^	Major depressive disorder (DSM-IV); HDRS	Lower depression prevalence and severity in intervention group
Maselko et al.^36^	PHQ-9; SCID	Sustained reduction in maternal depression up to 36 mo postpartum
Rahman et al.^37^	MDE remission (SCID)	Non-inferior to standard THP; higher remission in digital peer-delivered group
Lund et al.^38^	HDRS response	No statistically significant difference between intervention and control
Smith Fawzi et al.^39^	PHQ-9	Baseline data only; effectiveness outcomes reported separately
Rafat et al.^43^	Depression score/PPD frequency	Significant reduction in depression and postpartum depression frequency
Holmes et al.^42^	Depression and anxiety scores	Significant reduction in depression and anxiety; improved family planning uptake

### Critical appraisal of included studies

In general, the methodological quality of the included studies ranged from moderate to high. Nearly all randomized and cluster-randomized trials demonstrated appropriate randomization procedures and baseline comparability between groups, with larger trials showing stronger rigor through the use of validated diagnostic instruments, intention-to-treat analyses, and blinded outcome assessment. On the other hand, blinding of participants and intervention providers was generally not feasible due to the behavioural nature of psychological interventions, contributing to potential performance bias. Smaller pilot and digital intervention studies exhibited higher risk of bias related to unclear allocation concealment, limited reporting, and small sample sizes. Attrition was noted in longer follow-up studies but was typically addressed analytically. The formative mixed-methods study focused on feasibility rather than effectiveness and therefore was not appraised using RCT criteria (Table 4).

**Table 4:** Clinical appraisal of included studies.

Study	Design	Methodological quality	Key strengths	Key limitations	Overall appraisal
Jannati et al.^32^	RCT	Moderate	Randomized design; validated EPDS; clear intervention protocol	Allocation concealment unclear; no blinding	Moderate quality
Abujilban et al.^33^	RCT	Moderate	Random allocation; controlled analysis; standardized IPT	Small sample; participant/provider blinding not feasible	Moderate quality
Yator et al.^34^	Pilot RCT	Moderate–Low	Task-shared model; feasibility demonstrated	Very small sample; limited power	Moderate–low quality
Rahman et al.^35^	Cluster RCT	High	Large sample; diagnostic interviews; strong implementation fidelity	Blinding not possible; cluster effects	High quality
Maselko et al.^36^	Cluster RCT	High–Moderate	Long follow-up; rigorous outcome measures	Attrition over 36 mo	High–moderate quality
Rahman et al.^37^	Cluster RCT	High	Large non-inferiority trial; blinded assessors; cost analysis	Participant blinding impossible	High quality
Lund et al.^38^	Cluster RCT	Moderate	Well-designed task-sharing model; validated tools	No significant primary outcome effect; cost-effectiveness unfavorable	Moderate quality
Smith Fawzi et al.^39^	Cluster RCT	Moderate	Large HIV-affected cohort; standardized mhGAP comparator	Baseline paper only; effectiveness reported separately	Moderate quality
Rafat et al.^43^	RCT	Moderate–Low	Digital innovation; randomized	Blinding and concealment unclear; limited reporting	Moderate–low quality
Holmes et al.^42^	Cluster RCT	Moderate	Integration into routine services; strong public health relevance	Possible contamination; outcome assessor blinding unclear	Moderate quality
Boisits et al.^40^	Mixed-methods	Not applicable (non-RCT)	Contextual adaptation; stakeholder engagement	No comparator; no clinical outcomes	Developmental study

### Feasibility and acceptability of interventions

Evidence on feasibility and acceptability outcomes were assessed across all included studies, although the approaches and emphasize differed. The majority of studies examined on the practical aspects of implementing task-shared psychological interventions, such as participants’ recruitment, deliver the intervention with existing resources, and integrates it into routine primary health care or community settings. Participants and stakeholders perspectives were commonly used to assess acceptability feedback, highlighting satisfaction with the intervention, perceived relevance, and cultural appropriateness. On the whole, brief task-shared counselling interventions delivered by community health workers were generally reported as feasible, safe, and well accepted. None of the studies reported intervention-related adverse events were documented, further supporting the safety and acceptability of these approaches. On the other hand, not all studies assessed feasibility and acceptability in the same depth, and some focused primarily on intervention delivery or implementation processes rather than systematically measuring these outcomes. In particular, a formative mixed-methods study in South Africa demonstrated that brief, task-shared counselling interventions delivered by community health workers were acceptable, feasible, and contextually appropriate. Stakeholders supported a three-session counseling model, emphasizing its compatibility with existing primary health care and community outreach systems. This study informed intervention design but did not assess clinical effectiveness**.**

## Discussion

This systematic review synthesized evidence on the effectiveness of psychological interventions for perinatal depression, with a particular focus on task-shared and community-delivered approaches in low- and middle-income countries (LMICs). The results indicates that psychological interventions can substantially reduce perinatal depressive symptoms, particularly when they are structured, culturally adapted, and embedded within existing primary health care systems. Importantly, our findings indicate that the effectiveness of task-sharing is highly dependent on the delivery framework. We observed that task-sharing models augmented by technology-assisted tools or peer-delivered lived experience consistently showed non-inferior or superior outcomes compared with standard models. Conversely, standalone task-shared counseling lacking digital decision support or robust systemic reinforcement produced mixed or null effects in certain settings, particularly when compared against enhanced usual care. Rather than contradicting the viability of task-sharing, these results suggest that while the strategy is fundamentally sound for addressing the treatment gap, its success is contingent upon specific contextual, implementation, and scalability “boosters” like digital fidelity tools or peer engagement.

### Evolution of psychological interventions for perinatal depression

The early evidence from different research on perinatal depression interventions was dominated by specialist-led, clinic-based psychotherapy models, primarily in high-income settings.^2^^,^^44^^,^^45^ Despite efficacy of the intervention demonstrated, it require intensive resource and poorly scalable, limiting their applicability to LMIC contexts.^17^^,^^21^ In LMICs, task-sharing models were developed to address critical shortage of mental health specialists, with early studies demonstrating that supervised lay or non-specialist providers could effectively deliver evidence-based psychological care.^23^^,^^24^^,^^46^ This intervention strategy laid the foundation for later perinatal mental health programs, prioritizing accessibility, affordability, and integration into routine maternal care.^3^^,^^47^ This review places its findings within this historical context, showing that task-shared psychological interventions have advanced from feasibility to comparative effectiveness and implementation optimization.

### Effectiveness of task-shared interventions

The most robust evidence in this review is derived from large, rigorously conducted cluster randomized trials, particularly those evaluating the Thinking Healthy Program (THP) and its adaptations that are consistent with earlier trials,^24^^,^^48^ the included studies show that CBT-based interventions delivered by non-specialists can achieve high remission rates for perinatal depression. Nevertheless, the heterogeneity in outcomes across settings is noteworthy. Despite the fact that the Pakistan-based THP trials demonstrated robust effectiveness, the South African task-sharing trial reported no significant advantage over enhanced usual care.^38^ Comparable variability has been observed in previous studies across Africa and South Asia.^25^^,^^36^^,^^49^ These differences may reflect contextual differences in health system capacity, supervision quality, baseline care levels, and participant vulnerability, rather than intrinsic limitations of task-sharing itself.^50^^,^^51^

### Role of digital and technology-assisted delivery

Evidence synthesized in this review suggests that technology assisted delivery can enhance the effectiveness and scalability of task-shared psychological interventions. Furthermore, the non-inferiority of peer-delivered, digitally supported THP compared with standard health worker delivery aligns with emerging evidence that digital tools improve intervention fidelity, standardization, and supervision efficiency.^1^^,^^19^^,^^52^ Earlier studies demonstrate that digital mental health interventions can overcome barriers related to training intensity, provider drift, and supervision shortages, particularly in resource-constrained settings.^53^, ^54^, ^55^ The present findings extend this literature by demonstrating that technology can safely support delivery by peers with lived experience, without compromising clinical outcomes. Importantly, this supports global calls for hybrid models that combine human relational care with digital decision support, rather than fully automated interventions.^14^^,^^56^

### Peer delivery and lived experience

The effectiveness of peer-delivered interventions observed in this review is consistent with a growing literature emphasizing the therapeutic value of shared lived experience in perinatal mental health care.^11^^,^^17^^,^^57^ Intervention delivered by Peers may mitigate stigma, strengthen trust, and improve engagement, particularly among socioeconomically disadvantaged women.^5^^,^^58^

Despite their promise, earlier peer-led models often faced challenges related to inconsistent delivery quality and limited supervision.^59^^,^^60^ Evidence from the digitally supported intervention suggests that structured digital guidance may mitigate these limitations, enabling peers to deliver complex psychological content with high fidelity.

### Limited effects on secondary outcomes

Despite improvements in depressive symptoms, secondary outcomes such as anxiety, functional disability, and social support showed limited or inconsistent effects. This mirrors findings from prior perinatal mental health trials, where symptom reduction does not always translate into broader functional recovery.^8^^,^^15^^,^^61^

Several explanations are plausible. First, structural determinants such as poverty, food insecurity, and intimate partner violence highly prevalent among participants may constrain functional improvements despite symptom relief.^4^^,^^30^^,^^62^ Second, intervention durations may have been insufficient to affect entrenched social and economic stressors.^63^^,^^64^ These findings underscore the need for integrated psychosocial and social protection approaches, rather than narrow symptom-focused interventions.

### Safety and acceptability

No intervention-related adverse events were reported across all included trails, reinforcing the safety of psychological interventions delivered by non-specialists during the perinatal period. Consistent with prior evidence CBT- and PST-based interventions pose minimal risk when appropriately supervised.^9^^,^^10^ Additionally, formative research indicates high acceptability among women, providers, and health system stakeholders, particularly when interventions are brief, home-based, and aligned with existing service structures.^40^^,^^65^

### Effectiveness and health system integration

Evidence regarding cost-effectiveness was inconsistent, with one trial reporting that task-sharing was not cost-effective relative to enhanced usual care. This contrast with earlier economic evaluations suggesting that non-specialist delivery can reduce costs.^21^^,^^23^ The observed inconsistencies likely result from differences in comparator conditions, as enhanced usual care in sometimes encompasses substantial psychosocial support.^38^ This highlights the importance of context-sensitive economic evaluations, rather than assuming universal cost savings from task-sharing.

## Strength and limitation of the evidence

Strength of the reviewed evidence includes large sample sizes, diagnostic assessments, and real-world implementation. Limitations encompassed heterogeneous outcome measures, short follow-up, and underrepresentation of adolescent mothers and humanitarian settings and the inclusion of protocols studies likely reflecting a field still transition from development to scale-up.

### Implication for policy and practice and future research direction

The findings support scale-up of task-shared, evidence-based psychological interventions for perinatal depression, particularly those integrating digital support and peer delivery. Policymakers should prioritize integration into routine maternal and child health services, supported by structured training and supervision systems.^21^^,^^50^ Future research should focus on: Long-term maternal and child outcomes**,** Comparative cost-effectiveness across delivery models**,** Integration with social and economic interventions and Implementation in fragile and humanitarian settings.

## Conclusion

In conclusion, this systematic review provides strong evidence that task-shared psychological interventions particularly when supported by digitally tools are effective, safe, and scalable for addressing perinatal depression in LMICs. These findings align with global mental health priorities and highlight the urgent need to integrate mental health care into routine maternal health services.

## CRediT authorship contribution statement

**Abdissa Boka:** Writing – review & editing, Writing – original draft, Visualization, Validation, Software, Resources, Investigation, Formal analysis, Data curation, Conceptualization. **Debela Gela:** Methodology, Data curation. **Jembere Tesfaye:** Writing – review & editing, Validation, Software, Formal analysis, Data curation.

## Appendix Supplementary materials

Image, application 1
