# Supplementary material for: Effectiveness of psychological interventions for reducing depressive symptoms among women experiencing perinatal depression: a systematic review
Source: AJOG Glob Rep. 2026 Jun 1;6(2):100654. doi: 10.1016/j.xagr.2026.100654 (PMC13253125; doi:10.1016/j.xagr.2026.100654)
Supplement: Supplementary file 1 [file mmc1.docx]

## Appendix: Characteristics of Included Studies

##### Randomized Controlled Trial

| **Author (Year)** | **Country** | **Setting / Context** | **Study Design** | **Participants (Key Characteristics)** | **Intervention (Provider & Mode)** | **Comparator** | **Outcomes Measured** | **Main Findings** |
| --- | --- | --- | --- | --- | --- | --- | --- | --- |
| Jannati et al. (2020) | Iran | Three primary health care centers, Kerman | Parallel-group RCT (non-blinded) | Postpartum women ≥18 years; ≤6 months postpartum; EPDS ≥13 (n=75) | App-based CBT “Happy Mom”; 8 weekly sessions; self-guided mobile app | Usual care / no app | EPDS | Significant reduction in depressive symptoms in intervention vs control (p<0.001) |
| Abujelban et al. (2023) | Jordan | Governmental maternity hospital | Prospective RCT | Pregnant women 24–37 weeks; age 18–42; EPDS ≥12 (n=100) | Telephone-based IPT; 7 individual sessions; therapist-led | Routine antenatal care | EPDS | Intervention group showed significantly lower EPDS scores post-intervention (p<0.001) |
| Yator et al. (2022) | Kenya | Urban primary care clinics, Nairobi | Pilot RCT | Young mothers with HIV; 6–12 weeks postpartum; age 18–24 (n=24) | Group IPT (IPT-G); 8 sessions; CHW-led, face-to-face | Waitlist control | EPDS; ART adherence | Significant reduction in depressive symptoms; intervention feasible and acceptable |
| Rahman et al. (2008) | Pakistan | Rural community-based primary care | Cluster RCT | Prenatal & postnatal women with depression; socioeconomically deprived (n=903) | Thinking Healthy Programme (CBT-based); LHW-delivered home visits | Enhanced routine care | DSM-IV diagnosis; HDRS; infant outcomes | Lower maternal depression and improved infant outcomes in intervention group |
| Maselko et al. (2021) | Pakistan | Rural villages | Cluster RCT | Pregnant women (with/without depression); mean age 26.7 (n=1,154) | THP Plus (enhanced CBT); CHW-delivered, long-term | Enhanced usual care | PHQ-9; SCID; child outcomes | Sustained reduction in maternal depression up to 36 months postpartum |
| Rahman et al. (2025) | Pakistan | Rural Rawalpindi district | Cluster RCT (non-inferiority) | Perinatal women ≥18; SCID-confirmed MDE (n=980) | THP-TAP: peer-delivered CBT supported by digital app | Standard WHO-THP (LHW-delivered) | SCID remission; PHQ-9; GAD-7; WHODAS | THP-TAP non-inferior; higher remission at 3 months; safe and scalable |
| Lund et al. (2020) | South Africa | Peri-urban primary care clinics | Cluster RCT | Pregnant women ≥18; isiXhosa-speaking; EPDS ≥13 (n=384 MITT) | Task-shared counselling (PST/CBT); CHWs | Enhanced usual care | HDRS; EPDS; WHODAS | No significant difference on primary HDRS outcome; not cost-effective |
| Smith Fawzi et al. (2020) | Tanzania | Urban RCH/PMTCT clinics | Cluster RCT | Perinatal women living with HIV; PHQ-9 ≥9 (n=742) | Group psychosocial intervention (PST + CBT); lay providers | Improved standard care (mhGAP) | PHQ-9; IPV; social support; ART adherence | Baseline results only; high vulnerability; effectiveness assessed later |
| Boisits et al. (2021) | South Africa | Community Health Centres & household | Formative mixed-methods study | Pregnant women with mild–moderate distress; CHWs & health workers | Brief task-shared counselling model; CHW-delivered | Not applicable | Feasibility; acceptability; design outcome | Developed feasible 3-session CHW-led counselling intervention |
| Rafat et al. (2025) | Iran | Prenatal clinics, teaching hospitals | RCT | Pregnant women attending prenatal care (n=108) | App-based health promotion intervention | Routine prenatal care | Depression score; PPD frequency | Reduced depression and PPD frequency; moderate risk of bias |
| Holmes et al. (2025) | Ethiopia | Health centers, West Gojjam Zone | Cluster RCT | Postpartum women aged 16–24; married (n=302) | Mothers Time: Group CBT by HEWs | Standard postpartum care | Depression; anxiety; FP use | Significant reduction in depression/anxiety; increased FP uptake |

**(Y = Yes, N = No, U = Unclear, NA = Not Applicable)**

JBI Questions

Q1 Randomization

Q2 Allocation concealment

Q3 Baseline similarity

Q4 Participant blinding

Q5 Personnel blinding

Q6 Outcome assessor blinding

Q7 Identical treatment other than intervention

Q8 Complete follow-up

Q9 Intention-to-treat

Q10 Same outcome measurement

Q11 Appropriate statistical analysis

Q12 Appropriate trial design

| **Study** | **Q1** | **Q2** | **Q3** | **Q4** | **Q5** | **Q6** | **Q7** | **Q8** | **Q9** | **Q10** | **Q11** | **Q12** | **Overall Risk** |
| --- | --- | --- | --- | --- | --- | --- | --- | --- | --- | --- | --- | --- | --- |
| **Jannati 2020** | Y | U | Y | N | N | U | Y | Y | Y | Y | Y | Y | Moderate |
| **Abujelban 2023** | Y | Y | Y | N | N | U | Y | Y | Y | Y | Y | Y | Moderate |
| **Yator 2022 (Pilot)** | Y | U | Y | N | N | U | Y | U | Y | Y | Y | Y | Moderate–High |
| **Rahman 2008** | Y | Y | Y | N | N | Y | Y | Y | Y | Y | Y | Y | Low |
| **Maselko 2021** | Y | Y | Y | N | N | Y | Y | U | Y | Y | Y | Y | Low–Moderate |
| **Rahman 2025** | Y | Y | Y | N | N | Y | Y | Y | Y | Y | Y | Y | Low |
| **Lund 2020** | Y | Y | Y | N | N | Y | Y | Y | Y | Y | Y | Y | Moderate |
| **Smith Fawzi 2020** | Y | Y | Y | N | N | Y | Y | Y | Y | Y | Y | Y | Low |
| **Boisits et al. (2021)** | N | U | Y | NA | NA | NA | Y | Y | Y | Y | Y | Y | Low |
| **Rafat 2025** | Y | U | Y | N | N | U | Y | U | U | Y | Y | Y | Moderate–High |
| **Holmes 2025** | Y | Y | Y | N | N | U | Y | Y | Y | Y | Y | Y | Moderate |
